# The geographic distribution of melanoma incidence in Massachusetts, adjusted for covariates

**DOI:** 10.1186/1476-072X-5-31

**Published:** 2006-08-02

**Authors:** Laurie M DeChello, T Joseph Sheehan

**Affiliations:** 1 Department of Community Medicine and Health Care, University of Connecticut School of Medicine, 263 Farmington Ave, Farmington, CT 06030-6325, USA

## Abstract

**Background:**

The aims of this study were to determine whether observed geographic variations in melanoma cancer incidence in both gender groups are simply random or are statistically significant, whether statistically significant excesses are temporary or persistent, and whether they can be explained by risk factors such as socioeconomic status (SES) or the percent of the population residing in an urban rather than a rural area. Between 1990 and 1999, 4774 female and 5688 male melanomas were diagnosed in Massachusetts residents. Cases were aggregated to census tracts and analyzed for deviations from random occurrence with respect to both spatial location and time.

**Results:**

Thirteen geographic areas that deviated significantly from randomness were uncovered in the age-adjusted analyses of males: five with higher incidence rates than expected and eight lower than expected. In the age-adjusted analyses of females, six areas with higher incidence rates and eight areas with lower than expected incidence rates were found. After adjustment for SES and percent urban, several of these areas were no longer significantly different.

**Conclusion:**

These analyses identify geographic areas with invasive melanoma incidence higher or lower than expected, the times of their excess, and whether or not their status is affected when the model is adjusted for risk factors. These surveillance findings can be a sound starting point for the shoe-leather epidemiologist.

## Background

This study presents analyses of Massachusetts Cancer Registry (MCR) melanoma incidence data for cases diagnosed from 1990 to 1999. The study is an observational epidemiological investigation but uses complex statistical methods to determine whether variations in observed rates may be considered random or whether they represent true excesses or depressions. In addition to spatial and space-time analyses, which were only age adjusted, the study also demonstrates the degree to which findings are affected by covariates such a socioeconomic (SES) status or urban/rural status. The results can be used by public health officials to plan programs in areas where they are needed, as well as to evaluate programs already in place.

There have been conflicting findings of how urban or rural residence affects melanoma cancer incidence in international studies. Aase et al examined the Norwegian Cancer Registry data from 1955 to 1989.[1] They found urban residence to be associated with high melanoma incidence. However, Wesseling et al analyzed the Coasta Rica cancer registry data from 1981 to 1993 and found excess melanoma incidence in rural areas.[2]

Harrison et al looked at three factors in the Surveillance, Epidemiology, and End Results registry data from 1973 to 1993.[3] The percent below the poverty line was found to have an inverse relationship with melanoma incidence. Percent with at least a high school degree was associated with elevated melanoma incidence. Median household income was not associated with melanoma incidence. In Washington State from 1974 to 1985, living in a census tract with a high SES was found to be significantly associated with malignant melanoma.[4]

Latitude, [5-8] sunburn history, [5,8-10] and tanning bed exposure [11-13] have been shown to be associated with melanoma, as well. The latitude difference in Massachusetts may not vary enough to make a difference in melanoma incidence. However, the shore-line is a prime area for increased sun exposure due to vacationing in the summer months by the state's residents. Misconceptions about the safety of tanning beds increase users' risk of melanoma.

## Results

### Poisson regressions

The Poisson regression of male melanoma incidence showed that both SES components, wealth and poverty, had increased risk of melanoma incidence with each category of higher SES. These SES component categories were statistically significant (p < 0.0001) predictors of melanoma incidence risk for males across the state. Although percent urban was not statistically significant (p = 0.8865) in the Poisson regression, which estimates the effects of percent urban for the state as a whole, percent urban did have effects in the spatial analyses so was retained as a covariate.

The Poisson regression of female melanoma incidence revealed a different pattern in relation to the second SES component, poverty. The first three categories of component two were not significantly different and so they were lumped together. The forth and fifth categories were also not significantly different, so they also were lumped together so that the second component had two categories. The first SES component had a similar trend as compared to the males where the risk of melanoma increased with each higher category of SES (p < 0.0001). The second component with two categories also showed that the higher category of SES had increased risk (p < 0.0001). Again, urbanicity was not statistically significant in the Poisson regression (p = 0.2981).

### Purely spatial analyses of males

The observed counts, relative risks (RRs), and p-values for all statistically significant high and low geographic areas from the purely spatial analyses of age-adjusted melanoma incidence for males can be found in Table 1. The high areas are numbered and low areas lettered in order of statistical significance based on the analysis without covariates. Subsequent purely spatial results of males use these corresponding numbers and letters for significant areas that are in approximately the same geographical area.

**Table 1:** Age-adjusted male invasive melanoma cancer statistics for the purely spatial analyses, Massachusetts, 1990–1999.

**High Areas**	1	2	3	4	5	6	7	
No Covariate adjustment								
Obs^1^	478	719	494	57	183	*	*	
RR^2^	1.62	1.38	1.46	2.26	1.49	*	*	
p-value	<0.0001	<0.0001	<0.0001	0.001	0.005	*	*	
Percent Urban adjustment								
Obs^1^	403	719	618	57	*	76	*	
RR^2^	1.51	1.37	1.36	2.35	*	1.80	*	
p-value	<0.0001	<0.0001	<0.0001	0.0003	*	0.038	*	
SES adjustment								
Obs^1^	358	564	*	*	*	*	*	
RR^2^	1.72	1.29	*	*	*	*	*	
p-value	<0.0001	0.0002	*	*	*	*	*	
SES & Percent Urban adjustment								
Obs^1^	173	*	*	*	*	*	282	
RR^2^	1.66	*	*	*	*	*	1.38	
P-value	<0.0001	*	*	*	*	*	0.003	

**Low Areas**	A	B	C	D	E	F	G	H

No Covariates adjustment								
Obs^1^	157	58	17	49	303	32	40	*
RR^2^	0.49	0.39	0.25	0.44	0.72	0.41	0.48	*
p-value	<0.0001	<0.0001	<0.0001	<0.0001	<0.0001	<0.0001	0.002	*
Percent Urban adjustment								
Obs^1^	157	58	17	275	420	*	21	*
RR^2^	0.52	0.39	0.25	0.72	0.75	*	0.40	*
p-value	<0.0001	<0.0001	<0.0001	<0.0001	<0.0001	*	0.008	*
SES adjustment								
Obs^1^	*	58	*	395	*	*	*	418
RR^2^	*	0.49	*	0.72	*	*	*	0.78
p-value	*	<0.0001	*	<0.0001	*	*	*	0.0008
SES & Percent Urban adjustment								
Obs^1^	*	54	*	418	*	*	*	418
RR^2^	*	0.49	*	0.80	*	*	*	0.79
P-value	*	<0.0001	*	0.015	*	*	*	0.001

^1^Obs = observed cases. ^2^RR = relative risk. *Cluster not significant for this analysis.

**Figure 1:**
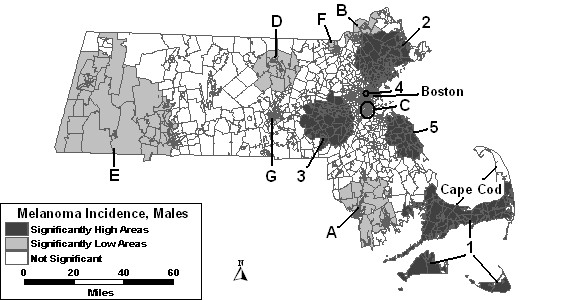
Purely spatial analysis for age-adjusted Massachusetts male invasive melanoma incidence, 1990–1999.

The purely spatial analysis of male melanoma incidence identified five areas of statistically significant excess and seven areas that had statistically significantly lower incidence rates. Figure 1 geographically displays the results of the purely spatial analysis without covariates. All areas of excess incidence were found in the eastern half of the state. The area with the most statistical significance is High 1 on Cape Cod, Nantucket, and Martha's Vineyard; it had a 62% higher incidence rate of melanoma than the rest of the state. Low A, west of Cape Cod, was the most statistically significant low area with a 51% lower incidence rate. The other 6 low areas are scattered around the state.

When urbanicity was added to the model as a covariate, High 1 covers slightly fewer tracts in the east and west compared to the model without covariates. However, it is still statistically significant along with high areas "2," "3," and "4." High 3 also changed shape to include a larger geographical area, and had a slightly lower RR. A new area became significant with this adjustment, labeled High 6, in the Boston area, while High 5 was longer statistically significant with the addition of urbanicity to the model. Low areas "A," "B," "C," and "G" remained stable despite this adjustment. Lows "D" and "E" increased in size, and Low D's RR increased to 0.72. Low F was no longer statistically significant with the adjustment.

When SES, both wealth and poverty components, were included as covariates in the purely spatial model, less than half of the areas remained significant when compared to the results of the model without covariates. High areas "1" and "2" remained statistically significant, but with diminished geographical size. All other areas of excess incidence were no longer statistically significant. Low B remained stable with the adjustment for SES compared to the analysis without covariates, while Low D increased in size, geographically. A new area appeared, labeled "H" in Table 1. All other low areas were no longer statistically significant.

**Figure 2:**
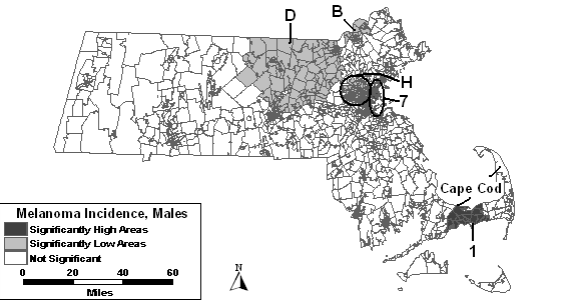
Purely spatial analysis for socioeconomic status- and urban/rural status-adjusted Massachusetts male invasive melanoma incidence, 1990–1999.

As seen in Figure 2, only two areas of excess incidence were found to be statistically significant when both SES and percent urban were entered as covariates in the purely spatial analysis of males. The same low areas were statistically significant as in the analysis adjusted for SES.

### Space-time analyses of males

The time frame, observed count, RR and p-value of all high and low areas from the space-time analyses of males can be found in Table 2. The numbers and letters of the high and low areas correspond approximately to the same geographical areas in the purely spatial analyses.

**Table 2:** Age-adjusted male invasive melanoma statistics for space-time analyses of Massachusetts, 1990–1999.

**Highs**	1	2	3	4	5	8	9			
Time frame	95–99	95–99	95–99	97–99	98–99	96–99	98–99			
No Covariate adjustment										
Obs^1^	360	474	469	54	44	*	*			
RR^2^	2.28	1.88	1.65	2.71	3.15	*	*			
p-value	<0.0001	<0.0001	<0.0001	0.0003	0.0002	*	*			
Percent Urban adjustment										
Obs^1^	378	474	452	54	185^3^	*	*			
RR^2^	1.99	1.86	1.63	2.80	1.61	*	*			
p-value	<0.0001	<0.0001	<0.0001	0.0004	0.001	*	*			
SES adjustment										
Obs^1^	369	403^6^	*	64	*	318	186			
RR^2^	2.22	1.78	*	2.36	*	1.44	1.78			
p-value	<0.0001	<0.0001	*	0.002	*	0.0003	<0.0001			
SES & Percent Urban adjustment										
Obs^1^	354^9^	356^2^	*	64	*	318	188			
RR^2^	1.85	1.71	*	2.42	*	1.48	1.80			
P-value	<0.0001	<0.0001	*	0.0004	*	<0.0001	<0.0001			

**Lows**	A	B	C	D	E	F	H	J	K	L

Time frame	90–99	90–98	90–97	90–95	90–96	91–99	91–92	90–91	90–91	90–96
No Covariates adjustment										
Obs^1^	157	47	60	64	154	26	79	*	*	*
RR^2^	0.49	0.36	0.43	0.44	0.52	0.37	0.56	*	*	*
p-value	<0.0001	<0.0001	<0.0001	<0.0001	<0.0001	0.002	0.010	*	*	*
Percent Urban adjustment										
Obs^1^	142^4^	47	60	164^5^	229	*	*	48	*	*
RR^2^	0.49	0.35	0.44	0.61	0.58	*	*	0.51	*	*
p-value	<0.0001	<0.0001	<0.0001	<0.0001	<0.0001	*	*	0.0497	*	*
SES adjustment										
Obs^1^	177^7^	*	12	254^5^	*	*	79^8^	46	42	203
RR^2^	0.64	*	0.29	0.64	*	*	0.47	0.41	0.43	0.70
p-value	0.0002	*	0.044	<0.0001	*	*	<0.0001	<0.0001	0.0002	0.029
SES & Percent Urban adjustment										
Obs^1^	176^7^	*	*	201	*	*	79^8^	37	41	*
RR^2^	0.64	*	*	0.68	*	*	0.48	0.35	0.43	*
P-value	<0.0001	*	*	0.001	*	*	<0.0001	<0.0001	<0.0001	*

^1^Obs = observed cases. ^2^RR = relative risk. ^3^The time frame for High 5 when adjusting for percent urban is 97–99. ^4^The time frame from Low A when adjusting for percent urban is 90–95. ^5^The time frame for Low D when adjusting for percent urban and SES separately is 90–96. ^6^The time frame for High 2 when adjusting for SES and SES with percent urban is 96–99. ^7^The time frame for Low A when adjusting for SES and SES with percent urban is 90–94. ^8^The time frame for Low H when adjusting for SES and SES with percent urban is 90–92. ^9^The time frame for High 1 when adjusting for SES and percent urban is 96–99. *Cluster not significant for this analysis.

**Figure 3:**
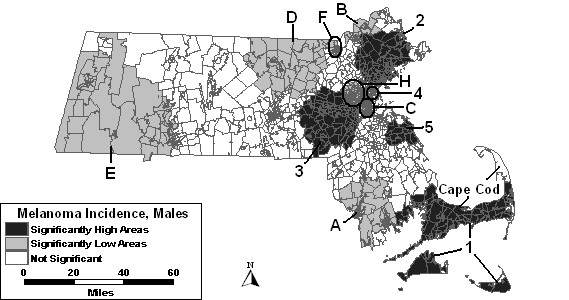
Space-time analysis for age-adjusted Massachusetts male invasive melanoma, 1990–1999.

Figure 3 displays the results of the space-time analysis of males without covariates. This analysis identified five areas of excess and seven areas of low incidence that were statistically significant. High 1 was significant for the last 4 years of the study period. High areas "2" through "5" varied slightly geographically from the areas of excess in the purely spatial analysis. They cover from 2 to 4 of the latest years of the study period. Low A was significant for the entire study period. Low areas "B" through "F" and area "H" were significant, while Low "G" was not.

The space-time analysis of males adjusted for percent urban found the same five areas of excess incidence to be significant as was found in the space-time analysis without covariates. However, High 1 and High 3 expanded in size geographically. High 5 was significant for an additional year of the study period with this adjustment. Low areas "B" and "C" remained stable with adjustment of percent urban. Low A was much smaller geographically and significant for a shorter time frame: 1990 to 1995. Low D included more tracts and was significant from 1990 to 1996. Low E also expanded geographically, but the time frame remained the same as in the analysis without covariates. Low areas "F" and "H" were no longer statistically significant after adjusting for percent urban. However, Low D covers the area that was included in Low F. A new area became statistically significantly low in the northeast corner of the state, labeled "J," which was significant for the first two years of the study period.

The space-time analysis of men adjusted for SES changed all high and low areas compared to the space-time analysis without covariates in either their statistics or coverage of geographic area. High areas "3" and "5" were no longer statistically significant. Two new areas became statistically significantly high: High 8 and High 9. What is surprising about High 9 is that it covers much of what had been low area E. However, the years for high area 9 are 1998 and 1999, and the years for Low E are 1990 to 1996. The statistics and/or time frame for Lows "A," "C," "D" and "H" were altered. Low areas "B," "E," and "F" were no longer significant when adjusting for SES. Two new areas became statistically significantly low when adjusting for SES. Low K overlapped a large geographic portion of High 9 and was significant for the first 2 years of the study period. Low L overlapped the northern portion of High 8 and was significant for the first seven years of the study period.

**Figure 4:**
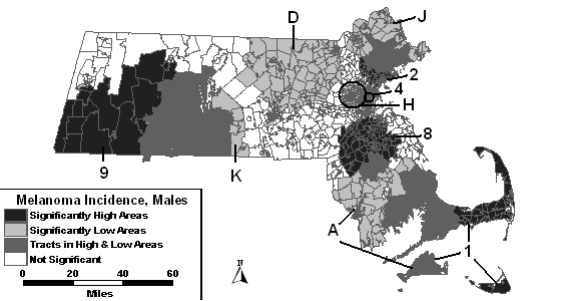
Space-time analysis for socioeconomic status- and urban/rural status-adjusted Massachusetts male invasive melanoma incidence, 1990–1999.

The space-time analysis of males adjusted for SES and percent urban together was similar to the results of the analysis adjusted for SES alone. Figure 4 depicts these results. Low areas "B," "C," "E," "F" and "L" were not statistically significant in the space-time analysis adjusted for SES and urbanicity.

### Purely spatial analyses of females

The observed count, RR, and p-value of each statistically significant area of excess or low melanoma incidence from the purely spatial analyses of females can be found in Table 3. The high areas are numbered and low areas are lettered in order of statistical significance based on the analysis without covariates with "1" and "A" being the most significant. Areas that covered approximately the same geographical area in the analyses with covariates as the analysis without covariates were given the same number or letter as the area in the analysis without covariates.

**Table 3:** Age-adjusted female invasive melanoma cancer statistics for the purely spatial analyses, Massachusetts, 1990–1999.

**Highs**	1	2	3	4	5			
No Covariate adjustment								
Obs^1^	682	322	238	100	87			
RR^2^	1.45	1.75	1.61	2.12	1.72			
p-value	<0.0001	<0.0001	<0.0001	<0.0001	0.049			
Percent Urban adjustment								
Obs^1^	671	315	358	93	*			
RR^2^	1.42	1.44	1.52	2.19	*			
p-value	<0.0001	<0.0001	<0.0001	<0.0001	*			
SES adjustment								
Obs^1^	476	405	*	123	*			
RR^2^	1.25	1.59	*	1.63	*			
p-value	0.012	<0.0001	*	0.008	*			
SES & Percent Urban adjustment								
Obs^1^	*	279	*	93	*			
RR^2^	*	1.42	*	1.74	*			
P-value	*	0.0004	*	0.015	*			

**Lows**	A	B	C	D	E	F	G	H

No Covariates adjustment								
Obs^1^	127	64	275	115	57	139	*	*
RR^2^	0.50	0.41	0.66	0.56	0.48	0.62	*	*
p-value	<0.0001	<0.0001	<0.0001	<0.0001	0.0002	0.0002	*	*
Percent Urban adjustment								
Obs^1^	132	64	281	*	57	24	343	*
RR^2^	0.53	0.44	0.68	*	0.50	0.40	0.72	*
p-value	<0.0001	<0.0001	<0.0001	*	<0.0001	0.001	<0.0001	*
SES adjustment								
Obs^1^	*	*	281	*	77	330	*	29
RR^2^	*	*	0.71	*	0.59	0.75	*	0.47
p-value	*	*	<0.0001	*	0.006	0.0003	*	0.045
SES & Percent Urban adjustment								
Obs^1^	*	10	252	*	*	19	*	*
RR^2^	*	0.26	0.74	*	*	0.39	*	*
P-value	*	0.001	0.005	*	*	0.016	*	*

^1^Obs = observed cases. ^2^RR = relative risk. *Cluster not significant for this analysis.

**Figure 5:**
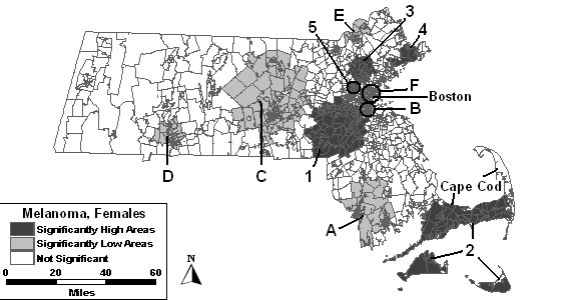
Purely spatial analysis for age-adjusted Massachusetts female invasive melanoma incidence, 1990–1999.

The purely spatial analysis without covariates of females identified 5 areas of excess incidence and 6 areas of low incidence to be statistically significant, as shown in Figure 5 and summarized in column 2 of Table 3. The high area with the most statistical significance was High 1, southwest of the Boston area with a 45% higher than expected incidence rate. The other 5 areas of excess cover much of the greater Boston area, as well as Cape Cod and the Islands. The most statistically significant low area, Low A, west of Cape Cod, had a RR of 0.50. Three significantly low secondary areas were in and around Boston, while 2 others were found in central and Western Massachusetts.

Percent urban added as a covariate to the purely spatial model of females found high areas "1," "2," "3," and "4" to remain statistically significant. High 5 was no longer statistically significant with this adjustment. Low areas "B" and "E" remained stable, while low areas "A," "C" and "F" changed in geographical area. Although Low D was no longer statistically significant itself, a larger area covering a large portion of western Massachusetts became significant, labeled Low G in Table 3, and included the geographical area of Low D.

The purely spatial analysis adjusted for SES found high areas "1," "2" and "4" to vary slightly from the analysis without covariates. High areas "3" and "5" were not statistically significant with this adjustment. Low C covered the same geographical area as found in the analysis adjusted for urbanicity. Low areas "E" and "F" increased in geographic area. Low areas "A," "B," and "D" were no longer significant. However, a new area, Low H, became statistically significantly low west of Boston.

**Figure 6:**
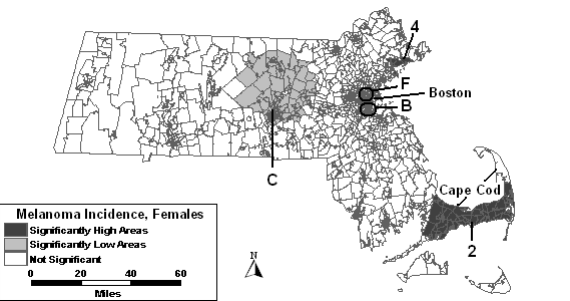
Purely spatial analysis for socioeconomic status- and urban/rural status-adjusted Massachusetts female invasive melanoma incidence, 1990–1999.

The purely spatial analysis where both SES and urbanicity, as seen in Figure 6, were included as covariates found only two areas to remain statistically significantly high: High 2 and High 4. Three areas of low incidence remained significant: low areas "C," "B" and "F," which all covered less geographic area compared to the analysis without covariates.

### Space-time analyses of females

The time frame, observed count, RR, and p-value of each statistically significant high or low area found in the space-time analyses of females can be found in Table 4. The high and low areas are numbered and lettered the same as the geographic areas found in the purely spatial analyses of females.

**Table 4:** Age-adjusted female invasive melanoma statistics for space-time analyses of Massachusetts, 1990–1999.

**Highs**	**1**	**2**	**3**	**4**	**5**	**6**	
Time frame	96–99	94–99	92–99	96–99	93–99	97–99	
No Covariate adjustment							
Obs^1^	371	305	*	293	161	189	
RR^2^	1.97	2.03	*	1.68	1.66	1.53	
p-value	<0.0001	<0.0001	*	<0.0001	0.001	0.027	
Percent Urban adjustment							
Obs^1^	367	245	499	114^7^	*	228^6^	
RR^2^	1.93	1.81	1.50	1.96	*	1.51	
p-value	<0.0001	<0.0001	<0.0001	<0.0001	*	0.003	
SES adjustment							
Obs^1^	306	305	*	238^8^	*	*	
RR^2^	1.68	1.99	*	1.67	*	*	
p-value	<0.0001	<0.0001	*	<0.0001	*	*	
SES & Percent Urban adjustment							
Obs^1^	306	202^11^	*	236^8^	*	*	
RR^2^	1.63	1.83	*	1.68	*	*	
P-value	<0.0001	<0.0001	*	<0.0001	*	*	

**Lows**	**A**	**B**	**C**	**E**	**F**	**G**	**J**

Time frame	90–97	90–99	90–96	90–97	90–95	90–96	90–92
No Covariates adjustment							
Obs^1^	114	64	187	87	146	178	58
RR^2^	0.47	0.41	0.59	0.54	0.60	0.57	0.53
p-value	<0.0001	<0.0001	<0.0001	0.0002	<0.0001	<0.0001	0.027
Percent Urban adjustment							
Obs^1^	108^4^	245^5^	187	87	†^6^	178	64
RR^2^	0.48	1.81	0.61	0.54	0.08	0.58	0.53
p-value	<0.0001	<0.0001	<0.0001	<0.0001	0.0004	<0.0001	0.005
SES adjustment							
Obs^1^	*	*	187	149^9^	74^10^	*	68
RR^2^	*	*	0.64	0.63	0.45	*	0.52
p-value	*	*	<0.0001	<0.0001	<0.0001	*	<0.0001
SES & Percent Urban adjustment							
Obs^1^	*	*	214	149^9^	74^10^	*	64
RR^2^	*	*	0.70	0.64	0.46	*	0.51
P-value	*	*	0.007	0.003	<0.0001	*	0.002

^1^Obs = observed cases. ^2^RR = relative risk. ^3^The time frame for High 7 when adjusting for percent urban is 95–99. ^4^The time frame for Low A when adjusting for percent urban is 90–98. ^5^The time frame for Low B when adjusting for percent urban is 90–98. ^6^The time frame for Low F when adjusting for percent urban is 90–91. ^7^The time frame for High 4 when adjusting for percent urban is 90–99. ^8^The time frame for High 4 when adjusting for SES is 97–99. ^9^The time frame for Low E when adjusting for SES and SES with percent urban is 90–95. ^10^The time frame for Low F when adjusting for SES and SES with percent urban is 90–93. ^11^The time frame for High 2 when adjusting for SES and percent urban is 95–99. *Cluster not significant for this analysis. † Number of cases too small to report for confidentiality reasons.

**Figure 7:**
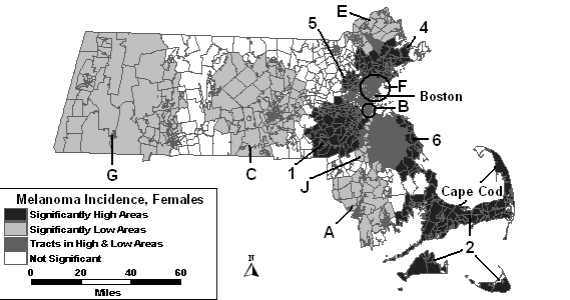
Space-time analysis for age-adjusted Massachusetts female invasive melanoma, 1990–1999.

The space-time analysis without covariates found five areas of excess melanoma incidence and seven areas of low incidence to be statistically significant, as shown in Figure 7. High 1 was significant for the last four years of the study period with 97% higher incidence than the rest of the state. High areas "2," "4" and "5" all increased in size geographically. High 3 was not statistically significant in the space-time analysis. Another area of excess, High 6, was found southeast of Boston. Low A covered more area in the space-time analysis for the first eight years of the study period. Low B was significant for the entire study period, and therefore had the same RR as in the purely spatial analysis. Low areas "C," "E" and "F" expanded, while Low G and a new low area, "J," were also significant.

When percent urban was added as a covariate to the space-time model, High 1, High 2 and High 4 were reduced in size compared to the space-time analysis without covariates. High 3 was statistically significant in this analysis from 1992 to 1999, whereas it was not significant in the analysis without covariates. High 6 reduced in size, but was significant for 2 additional years compared to the analysis without covariates. High 5 was no longer statistically significant with adjustment for urbanicity. Low areas "C," "E," and "G" remained stable with adjustment. Low areas "A," "B," "F" and "J" were also significant, but with slight changes to geography or time frame.

The space-time analysis adjusted for SES found only three high areas remained statistically significant: high areas "1," "2" and "4." All other high areas were no longer significant with adjustment for SES. Low areas "C," "E," "F" and "J" remained significant, while low areas "A," "B," and "G" were no longer statistically significant with this adjustment.

**Figure 8:**
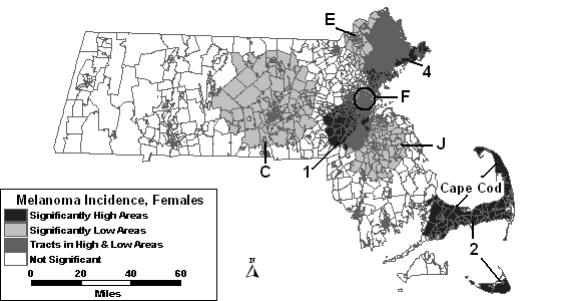
Space-time analysis for socioeconomic status- and urban/rural status-adjusted Massachusetts female invasive melanoma incidence, 1990–1999.

When SES and percent urban were added to the space-time model together, results were similar to the SES adjusted analysis. The results of these analyses can be seen in Figure 8. High areas "1," "2" and "4" were the only significant areas of excess. Low areas "E" and "F" were identical to the SES adjusted analysis, while low areas "C" and "J" were significant with slight changes.

## Discussion

Melanoma incidence followed similar geographic patterns in males and females between 1990 and 1999. Those living in geographic areas around Boston were at higher risk of melanoma, and those in the western and central portions of the state were at a much lower risk than the rest of the state. Males had a high risk of melanoma around the Boston area for the last five years of the study period or fewer years, whereas females had high incidence in the same general area for a longer period of time: the last four to ten years of the study period. Cape Cod, Nantucket, and Martha's Vineyard also had high incidence for both males and females for the last five and six years, respectively, and more than twice the expected number of cases. Many of the low areas were more stable than the areas of excess incidence. The most stable low area in the analysis of males was Low A, west of Cape Cod; it was significant for all 10 years of the study period. This area was also fairly stable for females as it was statistically significant for the first nine years of the study period.

Urbanicity did not explain the geographic variations in male melanoma incidence as much as SES. The purely spatial analysis with urbanicity as a covariate only removed one area of excess incidence as no longer significant and one low area, whereas only two high areas and two of the original low areas remained significant after adjustment for SES. Adjusting for urbanicity and SES together removed an additional high area. SES was also more influential in the female analyses in explaining why some areas were higher or lower in melanoma incidence. Urbanicity removed a high and low area, whereas SES removed two high and four of the original low areas. Adjustment for urbanicity and SES together only found two of the original high areas and three low areas to be statistically significant. These areas that disappeared with adjustment might be able to be assessed as to why the SES status and urban factors make these areas statistically significantly higher or lower risk than the rest of the state.

The current study adjusted for age, SES, and urban/rural status. Other known risk factors, such as sun exposure [5-10], could be used to possibly explain the high areas that were uncovered. Vacationing along the shoreline by Massachusetts residents during the summer may be a contributing factor for melanoma due to increased sun exposure. A meta-analysis of risk factors for melanoma [5] found a positive association with sun exposure that is intermittent and an inverse association to a more continuously high exposure to the sun. UV exposure from tanning beds also increases the risk of melanoma.[11-13] The following attributable risk percentages of other risk factors have been reported in the literature: 34.6% due to the Asp84Glu variant of the melanocortin 1 receptor[14], 10% to 38% due to family history[15], and 13.8% for men and 16.7% for women due to educational level, which is related to SES[16]. A significant association between BRCA2 and excess melanoma has also been reported [17].

### Limitations

Koh and colleagues found that cutaneous malignant melanoma was greatly underreported in Massachusetts between 1982 and 1986.[18] They estimated the underreporting to be about 12% with the possibility of being as high as 19%. There was not a significant time trend for the underreporting, so reporting did not appear to improve over time. There has not been a study published since to examine if this high percentage of underreporting continued. Therefore, if such a high rate of underreporting continued into the 1990's, it may have biased the current study.

Addresses are contracted out by MCR to companies that geocode them into their census tracts. Addresses that are geocoded into the wrong tract could potentially create smaller areas of statistically significant excess. This is especially problematic with smaller populations or with addresses such as a nursing home facility where more cases are likely to come from.

Post office addresses were not geocoded to a census tract. These cases were either put into the one tract that the town of the post office is part of, or were randomly assigned to tracts within that town. Most post office addresses occur in large cities. However, since cities have more cases compared to medium or smaller towns, a few post office address cases are not going to determine if a cluster is statistically significant or not.

Patients' usual residential address is used by MCR because it is easily obtained from patient medical records. However, this type of address is only representative of where they resided when they were diagnosed and may not be the same address where possible carcinogenic exposure occurred. Other possibilities include a former residence and occupational exposure, as well as the influx of population on Cape Cod and the Islands during the summer month, who may live elsewhere in the state the rest of the year or in other states.

## Conclusion

It is important for epidemiologists and public health officials to understand the epidemiology of melanoma incidence in their state in order to effectively target areas and populations with their limited resources for prevention and screening programs. This study helps to answer the questions of "Where?" and "Who?" in order to fine tune their programs. This study used age, urban/rural status and SES as covariates. Other covariates would be interesting to use in these types of analyses to further fine tune their programs.

Residents who are vacationing on Massachusetts beaches may have an increased risk of melanoma. Also, those with a higher SES are more likely to vacation in locations of increased sun exposure. Therefore, in looking at the results of this study, areas of excess that disappear after adjustment for SES should be targeted with prevention and screening programs. Areas of excess incidence that disappear or reduce after adjustment for SES should also be targeted for their lower SES population since screening programs may not be reaching this population. Tanning bed exposure may also be related to SES. Regulations regarding tanning beds are in place in Massachusetts [19], and modifications to these regulations have been proposed.

Urban/rural status of tracts might be linked with sun exposure practices and screening availability, as well. People living in urban areas may get more intermittent exposure to the sun, whereas residents in more rural tracts may have a more constant high exposure to the sun, but protect themselves from the exposure. Screening programs may be more readily available in urban areas as opposed to more rural areas. Minority populations are more likely to live in urban areas in Massachusetts. Therefore, the low areas that disappear with urban/rural status adjustment may be areas where screening needs to be increased, and high areas that disappear are where prevention programs targeting minorities need to be implemented.

The remaining areas of excess melanoma incidence after all adjustments should be examined by epidemiologists to determine what factors are driving the incidence rates in those areas. Also, the information provided in this report can be used to assess current programs in place to consider if they are working or if they need to expand their efforts. Areas that have significantly low incidence may either have prevention programs that are getting the message across, or they may be lacking in screening programs. Education programs regarding sun exposure should start as early in life as possible to reduce the risk of melanoma.

## Methods

Cases from the MCR included 4774 female and 5688 male invasive melanomas diagnosed from 1990 to 1999. The record for each case included date of diagnosis, gender, age, address and census tract of residence at the time of diagnosis.

Census tracts were the units for geographical aggregation. About 6.9% (n = 722) of the cases were not assigned to a census tract by MCR due to inaccuracies or omissions in the address of residence provided to MCR by either laboratories or doctors' offices. Therefore, we used town boundaries to randomly assign cases to a census tract within the town of residence, or town of their mailing address if a residential address was not provided. Town and tract boundary files were overlapped, and for towns completely contained in one tract, cases from that town were assigned to that tract. This method was used for 289 cases. For a town containing two or more census tracts, the cases missing tracts were randomly assigned to tracts within the town based on the proportion of the town's population each tract contributed. This was done using Microsoft Excel.[20] "RAND" function to create a three-digit random number between 0 and 999 for each case needing tract assignment. Each tract within a town was assigned a proportion of the numbers between 0 to 999 equal to the proportion of the town's population it accounted for. The case random numbers were then matched to the three-digit number assigned to a tract within the town. Only 433 (4.1%) of the cases had to be randomly assigned to a tract.

The socioeconomic status (SES) of each tract was used as a covariate in some of the analyses. An SES index was created based on the method of Yost et al using principal component analyses with varimax rotation[21]. Two components accounted for about 80% of the covariation among seven SES indicators from the 1990 Decennial Census[22]. The variables in the first component were median household income, median rent, median house value, and percent with at least a high school diploma and explained 49.1% of the variance. The variables in the second component included the percent unemployed, percent working class, and percent below the poverty level and explained 31.0% of the variance. Two component scores were computed for each census tract and these scores were divided into 5 equal categories; these categories were included in a Poisson regression to determine direction and statistical significance across the state for each gender separately.

The urban/rural status of each census tract was obtained from the U.S. Census Bureau from the 2000 Decennial Census.[23] The percent of each tract that was urban was calculated from the census data. This was chosen instead of a purely urban or rural designation since it provided more information. The percent urban of each tract was used as a covariate in some of the analyses.

Population count data for each census tract are from the 1990 [22] and 2000 [23] Decennial Censuses. Since some tracts in the early 1990s were later divided into multiple tracts in the late 1990's, tracts that changed between 1990 and 2000 were converted back to the tracts that existed in 1990 for this study.

### Spatial analyses

Age adjustments were done using Poisson regression to account for the population in each tract, separately for males and females. A weighted average of the 1990 and 2000 population counts by tract and age group were calculated; the natural log of the weight average population data was entered as the offset variable in the Poisson regression. The regression included age as a class variable and the number of cases in each tract and age group as the dependent variable. The resulting age-adjusted expected counts by tract were then used in the purely spatial and space-time analyses in place of the population counts.

To perform the purely spatial and space-time analyses, the SaTScan software[24] was used, which assumes that melanoma incidence follows a Poisson distribution. The null hypothesis of this model is that the probability of melanoma cases being diagnosed in a tract is equal throughout the state based on the population density. Further explanation of the spatial scan statistic and SaTScan can be found in Kulldorff et al, 1997 *Communications in Statistics *article [25].

The purely spatial and space-time analyses were performed separately by gender. The maximum spatial cluster size was set first at 25% of the population to test for excesses and deficits together, and then set at 10% to test for excesses and deficits separately making it possible for the same geographic area to show both excesses and deficits at different times within the same study period. This was performed to look for smaller, more meaningful areas of excess compared to a higher maximum spatial test size. Each of the geographic areas identified had a likelihood associated with it that was compared to the 9,999 likelihoods from the initial 25% maximum spatial size test. For the space-time analyses, the maximum temporal size was set at 90%. However, purely spatial clusters were also assessed, which ignore the temporal dimension, aggregating all years of the study period. The results of the 10% spatial maximum analyses are presented in the results section. Each gender was analyzed without covariates and then with percent urban, then wealth and poverty, then all covariates together in purely spatial and space-time analyses. The areas with the highest statistical significance in each analysis are the primary clusters. Statistically significant (p-value < 0.05) secondary areas are also presented since they were not likely to have occurred at random.

## Authors' contributions

LD: carried out data analyses and drafted the manuscript. TJS: PI, responsible for design, funding, of project with overall responsibility for implementing the project and participated in drafting the manuscript. All authors read and approved the final manuscript.
